# Spontaneous loss of B lineage transcription factors leads to pre-B leukemia in *Ebf1*^*+/–*^*Bcl-x*_*L*_^*Tg*^ mice

**DOI:** 10.1038/oncsis.2017.55

**Published:** 2017-07-10

**Authors:** J A Ramírez-Komo, M A Delaney, D Straign, K Lukin, M Tsang, B M Iritani, J Hagman

**Affiliations:** 1 Department of Comparative Medicine, University of Washington, Seattle, WA, USA; 2 Department of Biomedical Research, National Jewish Health, Denver, CO USA; 3 Department of Immunology and Microbiology, and University of Colorado Cancer Center, Colorado University Anschutz Medical Campus, Aurora, CO USA

## Abstract

Early B-cell factor 1 (EBF1) plays a central role in B-cell lineage specification and commitment. Loss of this critical transcription factor is strongly associated with high-risk, relapsed and therapy-resistant B–cell-acute lymphoblastic leukemia, especially in children. However, *Ebf1* haploinsufficient mice exhibit a normal lifespan. To determine whether prolonged survival of B cells would enable tumorigenesis in *Ebf1* haploinsufficient animals, we generated *Ebf1*^*+/–*^*Bcl-x*_*L*_^*Tg*^ mice, which express the anti-apoptotic factor Bcl-x_L_ in B cells. Approximately half of *Ebf1*^*+/–*^*Bcl-x*_*L*_^*Tg*^ mice develop aggressive oligoclonal leukemia as they age, which engrafts in congenic wild-type recipients without prior conditioning. The neoplastic cells display a pre-B phenotype and express early developmental- and natural killer cell/myeloid-markers inappropriately. In addition, we found tumor cell-specific loss of several transcription factors critical for maintaining differentiation: EBF1, TCF3 and RUNX1. However, in the majority of tumors, loss of *Ebf1* expression was not due to loss of heterozygosity. This is the first spontaneous mouse model of pre-B leukemia to demonstrate inappropriate expression of non-B-cell-specific genes associated with loss of *Ebf1, Tcf3* and *Runx1* expression.

## Introduction

*E*arly *B*-cell *f*actor 1 (EBF1) is an important transcriptional regulator of early B-cell development. In synergy with PAX5, EBF1 controls specification of progenitors to the B-cell lineage and maintains lineage commitment throughout development (reviewed in Ramirez *et al.*^1^). *Ebf1* gene expression begins in common lymphoid progenitors and increases as B-cell maturation proceeds, with the exception of terminally differentiated plasma cells.^2, 3^ In the absence of EBF1, B-cell development is arrested at the common lymphoid progenitor stage and functional B cells are not generated.^4, 5^

Loss of lymphoid and B-cell-specific transcription factors including IKZF1 (Ikaros), PAX5 and EBF1 are strongly associated with human B-cell-acute lymphoblastic leukemias (B-ALL, reviewed in ref. 6). Although *EBF1* mono-allelic deletions occur in a small fraction (4%) of total B-ALL cases, 25% of relapsed pediatric B-ALL patients carry *EBF1* mutations and *EBF1* deletion is strongly associated with a low relapse-free survival rate.^7, 8^ Tumors from high-risk leukemia patients such as those with *BCR-ABL1* translocations or *TP53/RB1* and *JAK* mutations are more likely to display *EBF1* haploinsufficiency than those from low-risk patients. Deletions in *EBF1* genes often interrupt the open reading frame, suggesting that loss of function contributes to disease progression and resistance to chemotherapy.^9^

EBF1 is a transcriptional activator, as well as a repressor.^10, 11, 12, 13^ EBF1 represses several natural killer (NK)/myeloid cell-specific genes, including *Cd244* (CD244; 2B4) and *Klrb1c* (NK1.1).^14^ These cell surface markers are expressed promiscuously in pro-B and early pre-B cells of *Ebf1* haploinsufficient mice, which also inappropriately expressed the early hematopoietic marker Sca-1 (*Ly6a*). However, aberrant expression of these factors was confined to specific B-cell subsets in the bone marrow and was not observed in developmentally progressed B cells in the periphery. Compared to wild-type mice, *Ebf1* haploinsufficient mice do not exhibit an increased incidence of tumors.^4, 15^

To investigate whether prolonging survival of pro-B cells would induce tumorigenesis, we crossed *Ebf1* haploinsufficient (*Ebf1*^*+/–*^) mice with transgenic mice (*Bcl-x*_*L*_^*Tg*^) overexpressing the antiapoptotic factor Bcl-x_L_ (*Bcl2l1*) specifically in B cells.^4, 16^ We report that a majority of *Ebf1*^*+/–*^*Bcl-x*_*L*_^*Tg*^ mice develop aggressive B-cell leukemia by slightly over one year of age. Development of disease is associated with significantly reduced expression of key transcription factors including EBF1, TCF3 (E2A) and/or RUNX1, which are critical for maintaining B-cell differentiation.^17^ Our results are consistent with the hypothesis that promoting survival of *Ebf1* haploinsufficient B cells results in tumorigenesis, likely after an accumulation of DNA damage and inactivation of critical transcription factors. The partial loss of cellular identity is manifested in these cells by aberrant expression of cell surface markers of NK, myeloid or early progenitor cells. These mice provide a useful new model for studying roles of EBF1 and the impact of its loss during leukemogenesis.

## Results

### Ebf1^+/–^Bcl-x_L_^Tg^ (EB) mice develop clonal lymphoproliferative disease

**Figure 1:**
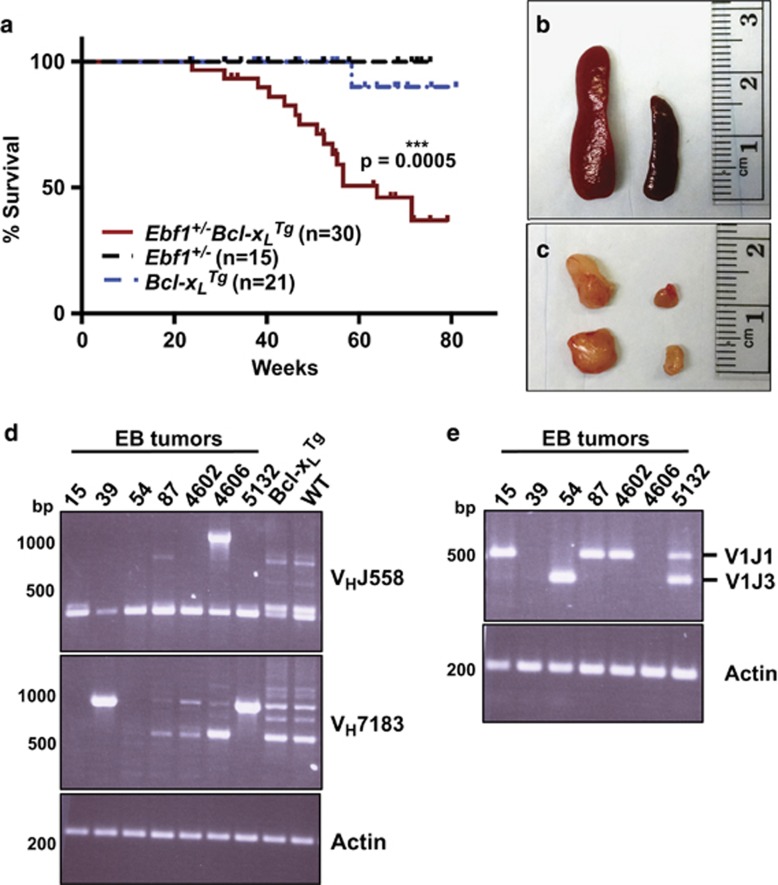
Ebf1^+/–^Bcl-x_L_^Tg^ (EB) mice develop clonal lymphoproliferative disease. (**a**) *Ebf1*^*+/–*^*Bcl-x*_*L*_^*Tg*^ (EB) mice display ~50% penetrance of lymphoproliferative disease (LPD). Compared to control littermates (black dashed; *Ebf1*^*+/–*^ and blue dashed; *Bcl–x*_*L*_^*Tg*^), EB mice (red) developed clinical disease. At week 80, fractions of affected mice were 15/30 (EB), 1/21 (*Bcl-x*_*L*_^*Tg*^) and 0/15 (*Ebf1*^*+/–*^). Affected mice displayed lethargy, hunched posture, enlarged lymph nodes and spleen. All aged *Ebf1*^*+/–*^and all but one *Bcl–x*_*L*_^*Tg*^ control littermates failed to develop clinical disease. *P*=0.0005 for EB vs *Bcl–x*_*L*_^*Tg*^ survival curves; Mantel–Cox test. *N* numbers given represent different mice of the indicated genotypes. (**b**–**c**) Comparison of spleens (**b**) and lymph nodes (**c**) from 1-year-old littermates: affected *Ebf1*^*+/–*^*Bcl-x*_*L*_^*Tg*^ (left), *Bcl–x*_*L*_^*Tg*^ control mouse (right). Spleen and lymph nodes from *Ebf1*^*+/–*^ and wild-type control animals appeared similar to the *Bcl–x*_*L*_^*Tg*^ control shown. Representative of 15 EB, 20 *Bcl–x*_*L*_^*Tg*^ and 15 *Ebf1*^*+/–*^ animals. (**d**–**e**) DNA was purified from affected *Ebf1*^*+/–*^*Bcl-x*_*L*_^*Tg*^ or control (*Bcl-x*_*L*_^*Tg*^, WT) lymph nodes and PCR was used to detect V_H_J558 or V_H_7183 immunoglobulin heavy chain rearrangements (**d**) and immunoglobulin lambda rearrangements (**e**). Each lane shows PCR product(s) from a different animal.

Mice heterozygous for an *Ebf1* knockout allele display a normal lifespan without overt disease development.^4^ In order to assess whether increasing the survival of B cells would allow *Ebf1* loss of heterozygosity to occur, we generated *Ebf1*^*+/–*^*Bcl-x*_*L*_^*Tg*^ (EB) mice that express high levels of the pro-survival factor Bcl-x_L_ under the control of the μ immunoglobulin heavy chain enhancer.^16^ These mice display a significantly shortened lifespan compared to control littermates, with a median survival of approximately 64 weeks of age (Figure 1a). Affected mice display hunched posture, lethargy, pale paws, ruffled hair coat, and enlarged spleen and peripheral lymph nodes including cervical, axillary, subiliac, colic and iliac nodes (Figures 1b and c). The tissues shown are representative of all control mice. PCR analysis of immunoglobulin heavy chain rearrangements (Figure 1d) and lambda light chain rearrangements (Figure 1e) in DNA isolated from the lymph nodes of seven different affected EB mice revealed monoclonal or oligoclonal cell populations. Most of these cell populations contained both light and heavy chain rearrangements, indicating that the progenitor cells had reached the pre-B, or later stages of B-cell development.

### EB mice develop aggressive disease with neoplastic infiltrates in multiple organs

**Figure 2:**
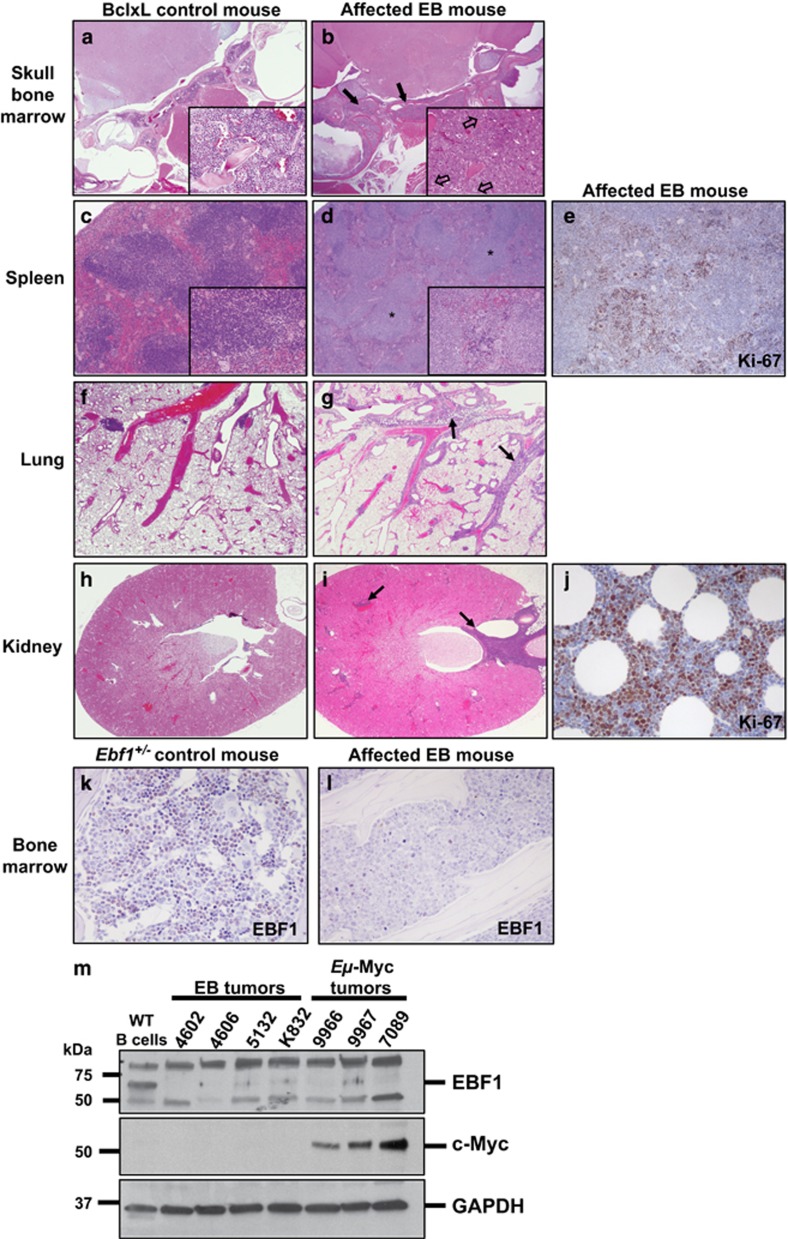
EB mice develop aggressive disease with neoplastic infiltrates in multiple organs. Compared to *Bcl-x*_*L*_^*Tg*^ control mice, affected EB mice have dense neoplastic round cell infiltrates within hematopoietic and lymphoid tissues including skull bone marrow (**a**, **b**) and spleen (**c**, **d**), as well as in the lungs (**f**, **g**) and kidneys (**h**, **i**). Insets show higher magnification. Within the skull (**b**) and some long bones (not shown), neoplastic cells fill and expand the medullary cavity. They also extend into the meninges and middle ear (**b**; black arrows). Neoplastic cells completely replace the hematopoietic tissue of the skull bones and are arranged in dense sheets among a scant fibrovascular stroma. Neoplastic cells are large, round to oval with scant to small amounts of eosinophilic homogenous cytoplasm and indistinct cell margins, while normal bone marrow displays a heterogenous population of cells (compare **b** and **a** insets). Nuclei are large, round to oval with vesicular chromatin and one to rarely two prominent central nucleoli. Anisokaryosis and anisocytosis are mild. Mitoses (**b**; open arrows) are frequent (8–10 per × 400 field). (**d**) Affected EB splenic white pulp (asterisks) is expanded by similar neoplastic cells yet maintains typical architecture. The red pulp contains hematopoietic cells (extramedullary hematopoiesis). Lungs (**g**) and kidneys (**i**) from EB mice have perivascular neoplastic infiltrates (black arrows), while *Bcl-x*_*L*_^*Tg*^ lungs (**f**) and kidneys (**h**) display limited infiltrates of small lymphocytes with a normal morphology. (**e**,**j**) Immunohistochemistry (IHC) shows neoplastic cells in the spleen (**e**) and mesenteric adipose tissue (**j**) which display intense nuclear staining with Ki-67 antibody. Images **a**–**j** are representative of three EB, three *Ebf1*^*+/–*^ and two *Bcl-x*_*L*_^*Tg*^ mice analyzed. (**k**, **l**) IHC reveals numerous cells in control bone marrow (**k**, *Ebf1*^*+/*−^) display nuclear staining with EBF1 antibody, while neoplastic cells in EB bone marrow do not retain stain (**l**). (**m**) Immunoblot showing EBF1 expression in whole cell extracts from wild-type MACS-sorted splenic B cells (EBF1 staining control) compared to whole cell extracts from pre-B tumor cell isolates in four different EB mice and three different Eμ-Myc mice. GAPDH is included as a loading control.

We performed histopathologic analyses of clinically affected EB mice as well as aged *Ebf1*^*+/–*^ and *Bcl-x*_*L*_^*Tg*^ littermates. Analysis of multiple organs including the medullary (bone marrow) cavities of the skull (Figures 2a and b), spleen (Figures 2c and d), lungs (Figures 2f and g) and kidney (Figures 2h and i) revealed dense accumulations of neoplastic round cells in EB mice (right panels), which were completely absent in *Bcl-x*_*L*_^*Tg*^ control mice (left panels). Within the bone marrow, spleen and lymph nodes, neoplastic cells almost completely effaced normal tissue parenchyma. Control mice displayed normal heterogeneous hematopoietic populations in the bone marrow of skull (Figure 2a inset) and long bones, in striking contrast to sheets of large cells with a lymphoblast morphology observed in the bone marrow of affected EB mice (Figure 2b inset). Ki-67 staining revealed many proliferating neoplastic cells within affected tissues, shown in the spleen (Figure 2e) and mesenteric adipose tissue (Figure 2j). Similar, but less abundant infiltrates of small lymphocytes with a normal morphology were observed in the lung, adipose tissue and kidney in aged *Bcl-x*_*L*_^*Tg*^, but not *Ebf1*^*+/–*^ control mice. These results are in accordance with published observations that *Bcl-x*_*L*_^*Tg*^ mice accumulate B cells in the periphery.^18^ None of the control littermates analyzed displayed clinical signs, gross or histologic evidence of lymphoproliferative disease. Immunohistochemical analysis showed EBF1 protein levels are reduced in neoplastic cells in the bone marrow of an affected EB mouse compared to normal bone marrow from an *Ebf1*^*+/–*^ control littermate (Figures 2k and l). To verify this finding we performed an immunoblot with whole cell lysates from four different primary EB tumors and utilized a different EBF1 antibody for detection. Lysate from wild-type splenic B cells was used as a positive control for EBF1 expression (Figure 2m, first five lanes, top panel). Loss of EBF1 protein was observed in other spontaneous pre-B-cell neoplasms, such as those that develop from B cells in transgenic *Eμ*-Myc mice, which overexpress cMyc (Figure 2m, last three lanes, top panel). Levels of cMyc protein in EB tumors are not highly elevated compared to those in *Eμ*-Myc pre-B tumors (Figure 2m, middle panel). This finding underscores the importance of loss of factors that maintain cellular identity during transformation, even in the context of different oncogenic drivers.

### Neoplastic cells display a precursor B-cell phenotype with aberrant expression of NK-cell markers

**Figure 3:**
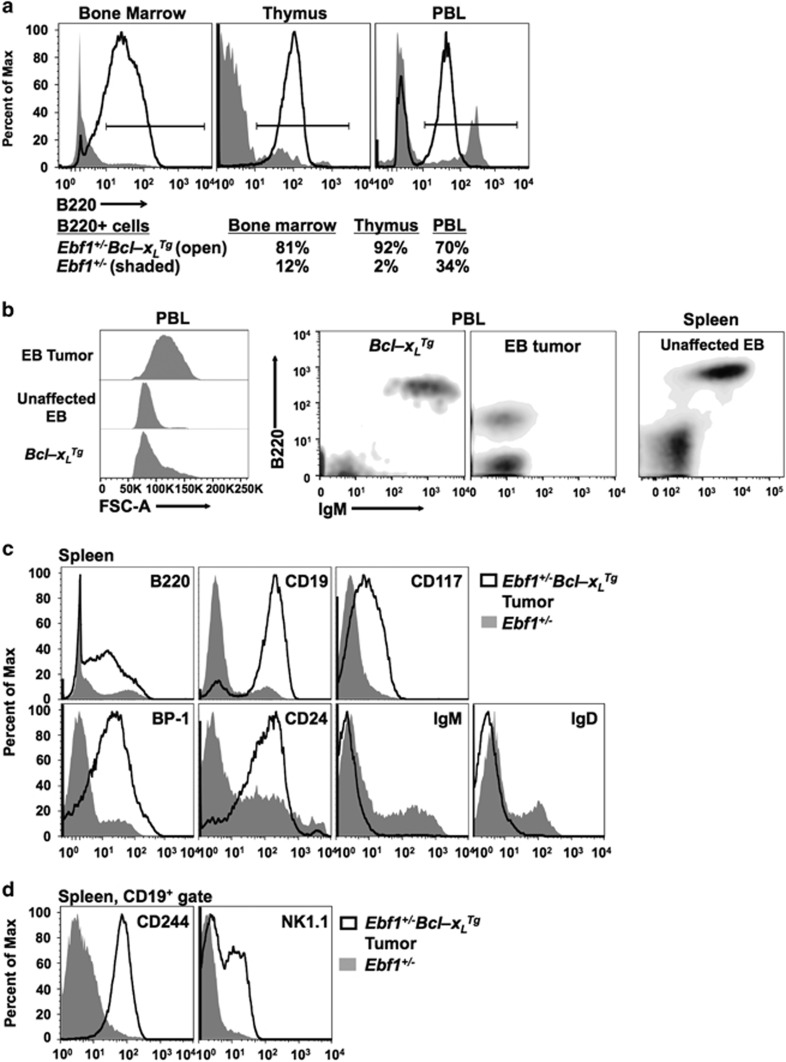
Neoplastic cells display a pre-B-cell phenotype with aberrant expression of NK-cell/myeloid markers. (**a**) Neoplastic cells in the bone marrow, thymus and peripheral blood lymphocytes (PBL) display a B-cell phenotype. B cells from 8-month-old littermate control (shaded) or EB (black line) bone marrow, thymus and PBL are shown. B-cell percentages are low in control mice due to their age. (**b**) Peripheral blood leukocytes (PBL) of affected EB but not unaffected EB or *Bcl-x*_*L*_^*Tg*^ control mice contain a uniform population of enlarged (lymphoblastic) B progenitor cells (B220^lo^IgM^−^). Unaffected EB mice do not display this population in the periphery (spleen). (**c**, **d**) Neoplastic cells in the spleen display an early B-cell progenitor phenotype with aberrant expression of the natural killer (NK) cell markers CD244 and NK1.1. Splenocytes from *Ebf1*^*+/–*^ or EB mice were labeled with the indicated antibodies for flow cytometry. All panels in (**b**, **c**) represent lymphocyte-gated cells. Panels in (**d**) are additionally gated on CD19^+^ cells. Neoplastic cells do not display membrane-bound immunoglobulin. Notably, CD19^+^ lymphoma cells display NK-cell markers as well as B-cell markers. Histograms and density plots in this figure are representative of eight affected EB, four unaffected EB, five *Bcl-x*_*L*_^*Tg*^ and five *Ebf1*^*+/–*^ control mice analyzed (see Table 1).

Flow cytometric analysis was performed on lymphoid tissues from EB mice as well as aged WT, *Ebf1*^*+/–*^and *Bcl-x*_*L*_^*Tg*^ littermates. Large populations of B220^+^ cells were observed in the bone marrow, thymus and peripheral blood leukocytes of affected EB mice (Figure 3a). Affected mice, but not control or unaffected EB mice, exhibited a uniform population of enlarged lymphoblastic cells in the peripheral blood (Figure 3b, far left panel) which displayed an early developmental B cell phenotype (B220^lo^IgM^–^, Figure 3b 3rd panel from left) and replaced populations of mature IgM^+^ B cells present in peripheral tissues of unaffected EB and control mice (Figure 3b, second and fourth panels from left). All leukemia cells analyzed displayed this B progenitor phenotype (Table 1). Primary isolates from seven different EB mice differed in BP-1 (*Enpep*) and CD25 (*Il2ra*) expression, with some tumors expressing BP-1 (present on late pro and early pre B cells) and others expressing CD25 (displayed on late pre B cells^19^) (Figure 3c, Table 1). All tumors examined expressed at least one of these pre-B-cell markers. Surface expression of CD117 (*kit*), an early developmental marker normally present on common lymphoid progenitors through early pro-B cells, was increased on most tumor cells (Figure 3c, Table 1). Notably, the NK/myeloid cell receptor CD244 (*Cd244*) and NK/T-cell protein NK1.1 (*Klrb1c*) were present on cells expressing the B-cell commitment marker, CD19 (Figure 3d). All tumor isolates displayed CD244 in large amounts, and most expressed low to moderate levels of NK1.1 (Table 1). However, tumor cells did not express CD335 (NKp46; *Ncr1*), a marker of active NK cells. Both NK1.1 and CD244 are aberrantly expressed on *Ebf1*^*+/–*^ pro-B and CD25^–^ pre-B cells.^14^ In contrast, several EB primary tumor cell isolates strongly displayed both CD25 and CD244, likely indicating they had progressed to the late pre-B stage before transformation occurred and CD244 expression was further upregulated. Aged *Bcl-x*_*L*_^*Tg*^ littermates did not display a population of cells with surface markers similar to the EB tumor cells (B220^lo^CD117^+^sIg^–^, Supplementary Figure S1). Neoplastic cells did not express markers characteristic of T cells or granulocytes (CD3, Ly-6G or CD11b, Table 1). The early developmental phenotype of the neoplastic cells, combined with a high abundance of lymphoblasts in the bone marrow and peripheral blood, suggest a precursor B-cell lymphoblastic lymphoma/leukemia derived from bone marrow precursors as the presumed tissue of origin.^20^

To discern whether EB tumor cells are transplantable, 2 × 10^6^ isolated lymph node cells from one of six different primary tumors were transferred to congenic immunodeficient recipient mice via intravenous injection (Table 2). The tumors that developed from these transfers were serially transferred through four to nine different cohorts of recipients, depending on the tumor isolate. All recipients of the tumor cells developed clinical disease within 2–12 weeks. To test whether EB tumors are aggressive enough to cause disease in immunocompetent recipients, 2 × 10^5^ cells from two different primary tumor isolates were transferred into healthy CD45.1 recipient mice without prior irradiation or conditioning. All recipients developed clinical disease within 4 weeks of injection. A distinct population of CD45.2^+^CD244^+^ cells was found in the peripheral blood, spleen, lymph nodes and bone marrow (Supplementary Figure S2).

### Pre-B lymphoma/leukemia is characterized by loss of lineage-commitment transcription factors

**Figure 4:**
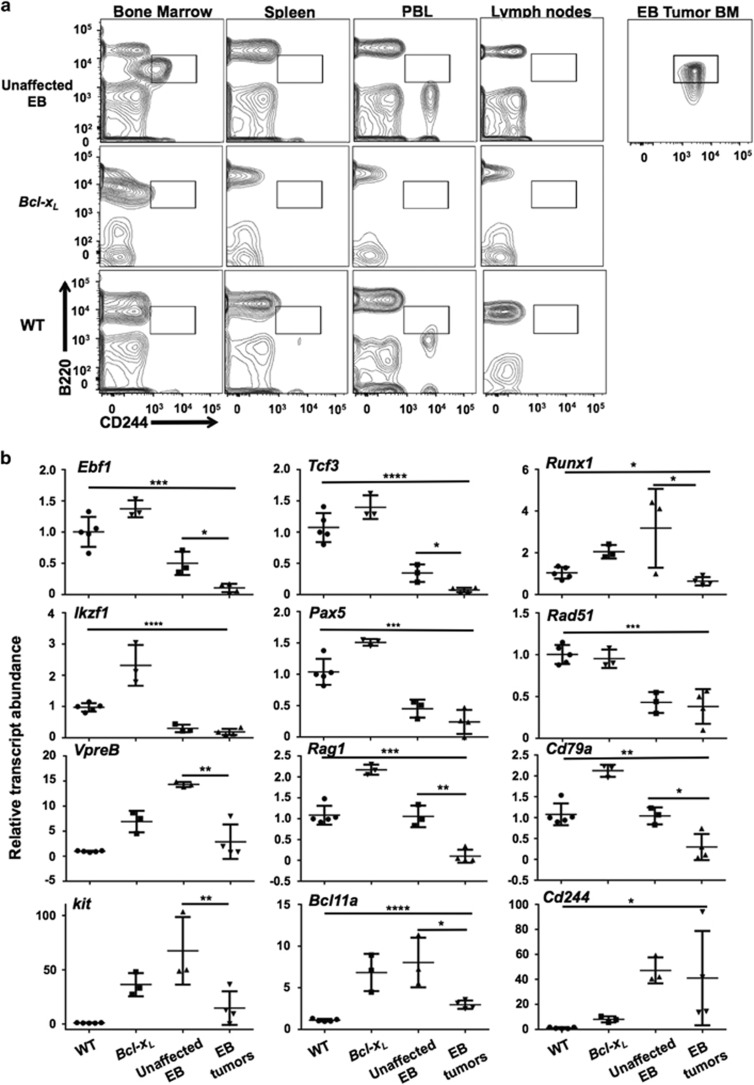
Pre-B lymphoma/leukemia is characterized by loss of lineage-commitment transcription factors. (**a**) A population of B220^lo^CD244^+^ cells is present in healthy EB mouse bone marrow but not in peripheral tissues or in *Bcl-x*_*L*_^*Tg*^ or wild-type mice. Additional staining (not shown) defined this population as late pre-B: B220^lo^CD19^+^BP-1^−^CD25^+^IgM^−^ (same phenotype as EB tumor cells, far right panel). Flow cytometric analyses of total bone marrow, splenocytes, peripheral blood leukocytes (PBL) and subiliac lymph nodes are shown. Fluorochromes used for B220 were PE-Cy7 and eFluor450. Contour plots in this figure are representative of eight affected EB, four unaffected EB, five *Bcl-x*_*L*_^*Tg*^ and five *Ebf1*^*+/–*^ control mice analyzed. (**b**) RT–qPCR analysis of relative transcript levels from selected genes in sorted late pre-B cells (B220^+^IgM^–^BP-1^–^CD25^+^) from the bone marrow of five congenic wild-type mice, three *Bcl-x*_*L*_^*Tg*^ mice, B220^lo^CD244^+^ (presumed tumor progenitor) cells from the bone marrow of three healthy EB bone marrow transfer recipients (Unaffected EB), and leukemic cells from four different EB mice (EB tumors). *P<0.05; **P<0.01; ***P<0.001; ****P<0.0001. Primers sequences are listed in Supplementary Table S1.

A unique population of pre-B cells in the bone marrow of healthy EB mice (Figure 4a, upper left panel) aberrantly expresses CD244. This population is characterized by B220^lo^/CD19^+^/IgM^−^ staining, includes CD25^+^ pre-B cells, and has also previously been observed in *Ebf1*^*+/–*^ mice.^14^ The population is not present in wild-type or *Bcl-x*_*L*_^*Tg*^ mice, or in peripheral tissues of healthy EB mice (Figure 4a). All EB tumors were B220^lo^/CD244^+^ (Figure 4a, far right panel, Table 1). Therefore, we hypothesized that this aberrant population comprises the progenitors of EB tumors. To investigate this possibility, total bone marrow from a healthy EB donor was transferred to four sub-lethally irradiated *Rag2*^−*/*−^*IL2rγ*^−*/*−^ recipients. All recipients were collected 16 weeks after transfer, when one bone marrow transfer recipient exhibited signs of clinical disease including abdominal effusion containing mononuclear round cells, splenomegaly and lymphadenopathy. This mouse (K832, Figure 4a far right panel, Table 1) displayed a single uniform population of B220^lo^CD19^+^IgM^–^CD25^+^CD244^hi^ cells in the bone marrow which largely effaced all other populations and matched EB tumor cell surface marker phenotype, suggesting that tumor cells developed from the unusual B220^lo^CD244^+^ late pre-B population present in EB and *Ebf1*^*+/–*^ bone marrow. In addition, sorted B220^lo^CD244^+^ populations from the bone marrow of these mice carry lambda light chain rearrangements similar to EB tumors (Supplementary Figure S3). Although mouse and human lymphatic tumors are commonly characterized based on cell surface expression of lineage-specific markers as well as distribution and molecular characteristics,^20, 21^ we cannot exclude the possibility that EB tumors arise from peripheral B cells.

To analyze the expression of genes important for B-cell development and lineage maintenance in EB tumor cells, we performed quantitative PCR analysis of cDNA from FACS-sorted wild-type and *Bcl-x*_*L*_^*Tg*^ BM late pre B cells (B220^+^IgM^–^BP-1^–^CD25^+^), healthy EB BM transplant recipients (B220^+^CD244^+^ BM; unaffected EB) and primary EB tumor isolates. Strikingly, significant reductions in *Ebf1*, *Tcf3* (encoding E2A), and *Runx1* transcripts were observed in tumor compared to unaffected EB cells (Figure 4b). Reductions of *Ebf1*, *Tcf3* and *Runx1* transcripts were also detected in EB tumor cells relative to lymph node cells from *Bcl-x*_*L*_^*Tg*^ control mice, confirming that levels of these transcripts are reduced even if the tumor cells are peripheral in origin (Supplementary Figure S4). Abundance of *Ikzf1* and *Pax5* transcription factor mRNA was not significantly different in tumor vs unaffected cells, but was markedly lower than in pre-B cells from wild-type and *Bcl-x*_*L*_^*Tg*^ mice. RUNX1, E2A, IKZF1 and PAX5 all positively regulate *Ebf1* transcription, and the *Pax5* gene itself is also positively regulated by EBF1 as part of a positive regulatory feedback loop. Mono-allelic deletions, point mutations and translocations of one or more of these transcription factors often occur in pediatric B-ALL.^9, 22^ Reduced levels of factors encoded by *Ikzf1*, *Runx1*, *Tcf3*, *Pax5* and *Ebf1* may each be important individually for predisposing EB mice to tumor development, while concerted loss of these factors, particularly in combination, would potently induce loss of differentiation. Transcript abundance of *Rad51* (encoding a common DNA damage response factor) was also significantly reduced in both tumor and unaffected EB cells compared to wild-type and *Bcl-x*_*L*_^*Tg*^ pre-B cells, in accordance with previous findings that *Ebf1* heterozygosity results in decreased expression of this gene and increased DNA damage in pro-B cells.^23^ Transcripts of the EBF1 target genes *VpreB, Rag1*, and *Cd79a/mb-1* were also significantly reduced in tumor cells compared to unaffected EB cells, likely as a result of reduced EBF1 activity.

Chromosomal translocations resulting in *BCL11A* overexpression are often seen in human B-ALL tumors, and *kit* is a proto-oncogene and early developmental marker upregulated in many hematopoietic neoplasms. Abundance of *Kit* (CD117) and *Bcl11a* transcripts were increased in unaffected EB cells compared to wild-type pre-B cells; however, this likely reflects increased *Bcl-x*_*L*_^*Tg*^ activity because *Bcl-x*_*L*_^*Tg*^pre-B cells displayed comparable levels of these transcripts. Additionally, we observed increased *Cd244* transcripts in tumor cells of EB mice alone, which correlates with increased cell surface display of CD244 observed in flow cytometric analyses. Putative EBF1 binding sites are present in the *Cd244* promoter and upstream regulatory module (JH, unpublished data), and EBF1 negatively regulates *Cd244* expression.^14^ We also examined *Irf4* and *Spi1* transcript levels as mice lacking expression of both *Spi1* (encodes PU.1) and IRF4 in B cells spontaneously develop pre-B leukemia.^24^ However, although levels of *Irf4* transcripts are significantly decreased in EB tumor cells, levels of *Spi1* transcripts were not significantly altered (Supplementary Figure S5). *Irf4* deficiency is not by itself associated with an increased risk of leukemia, but cooperates with oncogenes to promote leukemogenesis.^25^

### Loss of heterozygosity is not required for loss of EBF1 expression in EB tumors

**Figure 5:**
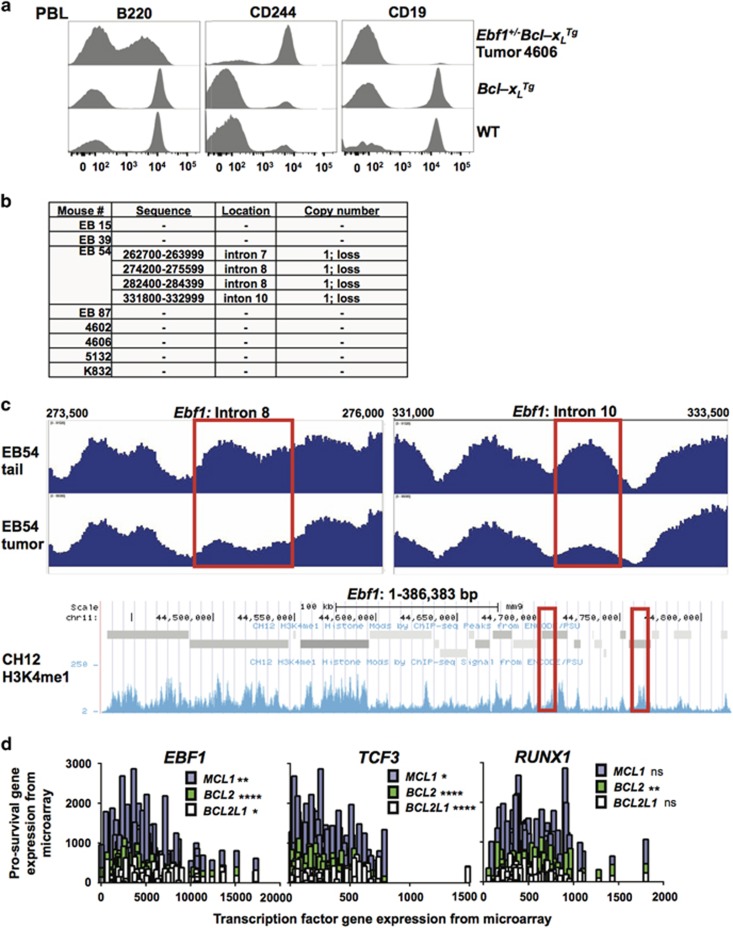
Loss of *Ebf1* expression does not occur through loss of heterozygosity in most EB tumors. (**a**) One EB tumor (4606) expressed B220 but not CD19, suggesting loss of Ebf1 expression and/or function in this tumor as CD19 is positively regulated by EBF1. Flow cytometric analysis of peripheral blood leukocytes (PBL) from aged controls and an affected EB mouse is shown. Tumor cells are B220^lo^/CD244^+^/CD19^–^. CD19 is a positive target and CD244 is a negative target of EBF1 transcriptional regulation. Histograms in this figure are representative of one affected EB, five *Bcl-x*_*L*_^*Tg*^ and five wild-type control mice analyzed. (**b**) High-throughput sequencing of *Ebf1* genes was performed on paired normal tissue (tail or ear clip) and tumor (lymph node or spleen) DNA from eight different mice bearing EB tumors. The table lists copy number variations in the samples found by the control-FREEC application and verified by visual inspection of aligned reads using normalized counts. One tumor (EB54) displayed ~50% loss of coverage inside four regions in introns 7, 8 and 10. (**c**) Histograms depicting depth of sequence coverage for two regions with loss in EB54 tumor compared to tail DNA are shown. Boxes highlight the areas of sequence coverage loss. ChIP-seq data show monomethyl H3K4 density across *Ebf1* in the CH12 murine B lymphoma cell line (Hardison PSU, UCSC accession wgEncodeEM002370). Boxes correspond to the locations of EB54 sequence coverage loss shown above. (**d**) Correlation of gene expression data for *EBF1*, *TCF3*, *RUNX1*, *BCL2L1*, *BCL2* and *MCL1* from *N*=177 high-risk pediatric B-cell-acute lymphoblastic leukemia (B-ALL) patient samples.^8^ The significance of each correlation is shown in the legend for each graph (*P<0.05; **P<0.01; ***P<0.001; ****P<0.0001, ns=not significant.) Data accessed from Gene Expression Omnibus (http://www.ncbi.nlm.nih.gov/geo), accession GSE11877.

One EB tumor, #4606, lacked surface expression of CD19 in contrast to all other tumors examined, which were CD19 positive (Figure 5a, Table 1). However, other cell surface markers of this tumor were identical to other EB tumors (B220^lo^/CD117^+^/CD25^+^/CD244^+^/sIg^–^). EBF1 and PAX5 coordinately activate CD19 expression, which is often used as an indicator of commitment to the B-cell lineage. Owing to this finding and the loss of EBF1 expression in EB tumors, we investigated whether loss of heterozygosity occurs in EB tumors similar to *EBF1* genetic lesions found in a subset of high-risk pediatric B-ALL. We performed high-throughput sequencing of *Ebf1* genes in paired DNA samples comprising EB primary tumor and tail tissue from each host (Figures 5b and c). Sequence analysis software was used to identify regions of change in *Ebf1* copy number; however, changes identified in repeat-rich areas with very low sequence coverage were not considered robust. *Ebf1* copy number loss occurred in introns 7, 8 and 10 in a single primary tumor, which was verified by qPCR analysis of these regions (data not shown). No other robust changes were observed in seven other primary tumors sequenced. ChIP-seq data from ENCODE/PSU shows that areas of *Ebf1* genes are enriched with monomethylated histone H3 lysine 4 in a murine B lymphoma cell line, with the regions of copy number loss in the EB54 tumor indicated (Figure 5c). This histone mark is enriched in genes poised for transcription, and often marks the location of enhancers. The areas of copy number loss identified in the EB tumor isolate fall within regions of increased H3K4me1 occupancy, so it is possible these areas might serve as enhancers. Overall, however, intragenic *Ebf1* lesions do not appear responsible for the observed decrease in *Ebf1* transcript levels in EB tumors.

Because DNA methylation frequently silences gene expression during both cellular differentiation and tumorigenesis, we investigated whether the *Ebf1α* (distal) and *Ebf1β* (proximal) promoters are methylated in EB tumors.^26, 27^ Transcripts from both promoters were significantly decreased in unaffected EB compared to EB tumors (Supplementary Figure S6). However, only transcripts from the *Ebf1β* promoter, the major source of *Ebf1* transcripts in B cells,^27^ were significantly decreased compared to wild-type pre-B cells. We next used bisulfite conversion of genomic DNA followed by PCR amplification and pyrosequencing to examine the methylation of CpGs 1–3 (distal-proximal) in the *Ebf1β* promoter and CpGs 15–22 (distal-proximal) in the *Ebf1α* promoter. However, these particular CpGs were largely demethylated in control non-lymphatic tissue (tail and ear clips) from EB mice, and methylation levels were significantly decreased in lymph node samples from all mice, both EB tumor and *Ebf1*^*+/–*^or *Bcl-x*_*L*_^*Tg*^ controls (data not shown). Other *Ebf1* promoter CpGs could be differentially methylated, or increased DNA methylation may not be responsible for the loss of EBF1 expression observed in EB tumors. Serial or concerted loss of Runx1 and E2A transcription factors, possibly through genetic lesions induced by DNA damage, might account for EBF1 loss. Since the distal *Ebf1β* promoter is much more active than *Ebf1α* and is regulated by Pax5, reduced expression of this factor may account for concurrent reductions in *Ebf1* transcripts. Accessibility and modification of chromatin in the *Ebf1* promoter and/or enhancers may also contribute, possibly through binding of transcriptional repressors and/or inhibitory chromatin remodelers such as Mi2-nucleosome remodeling and deacetylase complexes.

To investigate whether EB mice recapitulate selected gene expression signatures found in human B-ALL, we correlated the expression of three pro-survival Bcl2 family members (MCL1, BCL2 and BCL2L1 [BCL-XL]) and three key transcription factors (EBF1, TCF3 and RUNX1) in samples from a study of 177 pediatric patients with high-risk B-ALL (Figure 5d).^8^ B-ALL tumors often exhibit expression of multiple pro-survival Bcl2 family members. We found that low expression of each of the transcription factors is correlated with higher *BCL2* expression; however, only low expression of *TCF3* and *EBF1* correlated with levels of *BCL2L1* and *MCL1* expression. Interestingly, *BCL2L1* expression most strongly correlated with low expression of *TCF3*, which of the three transcription factors examined is also the most profoundly deficient in EB tumors. We also compared *PAX5, IKAROS* and *IRF4* expression with expression of the pro-survival proteins, and found no significant correlation except between *IKAROS* and *BCL2L1* (results not shown). Therefore, EB mice may serve as an appropriate model for precursor B-ALL since they exhibit multiple deficiencies in lineage determinant transcription factors and concurrent upregulation of one or more Bcl2 family members.

## Discussion

Leukemia is often thought to arise due to multiple lesions, or ‘hits’, including (1) loss of genes that mediate hematopoietic differentiation and (2) mutations that confer proliferative and/or survival advantages.^28^ Our observations suggest that loss of *Ebf1* genes is unlikely to be a primary driver of leukemogenesis by itself, but can synergize with mutations in other genes that increase the survival and relative fitness of leukemic cells. Heltemes-Harris *et al.*^15^ described two murine models of B-ALL characterized by *Ebf1* or *Pax5* haploinsufficiency and constitutive STAT5 activation (CaStat5). They also investigated whether extending survival of *Ebf1* haploinsufficient cells affected leukemogenesis by generating *Ebf1*^*+/–*^*Bcl-x*_*L*_^*Tg*^ (EB) mice, but did not report tumor development within ~250 days (36 weeks) of age. Our findings are compatible with these results, as by 36 weeks of age only 2/30 (6.7%) of our EB mice had developed tumors. The substantial increase in tumor incidence in CaStat5 mice as a result of *Ebf1-* or *Pax5*-haploinsufficiency underscores the importance of these transcription factors in maintaining cellular differentiation despite strong oncogenic signaling. In accordance, the Sigvardsson laboratory described similar development of pre-B leukemia in a subset of *Ebf1*^*+/–*^*Pax5*^*+/–*^mice with high molecular heterogeneity between tumors.^23^ Based on cell surface marker expression and analysis of transcripts of various B-cell and stage-specific factors, it appears pre-B tumors derived from EB mice display similar molecular heterogeneity. These observations lend support to the hypothesis that tumor development in the context of lineage-determinant transcription factor haploinsufficiency is a multistep process.

Translocations involving the key B lineage transcription factors RUNX1 (*ETV6-RUNX1*), TCF3 (*TCF3-PBX1*) and EBF1 (*EBF1-PDGFRβ*) have all been described in human B progenitor leukemias, and deletions affecting these loci frequently occur in high-risk pediatric B-ALL.^8^ Our finding of greatly reduced levels of these transcription factors in EB tumors compared to late pre-B cells and cells in peripheral lymph nodes indicates that further loss of lineage-determinant transcription factors in a haploinsufficient context potentiates tumorigenesis and/or transformation. Tumor development in EB mice likely begins with an accumulation of DNA damage due to reduced *Ebf1* dosage, combined with increased cell survival due to high levels of the antiapoptotic BCL-2 family member BCL-X_L_. Similar to BCL2, BCL-X_L_ synergizes with c-Myc to drive B progenitor leukemia.^29^ However, BCL-2 family members do not always serve redundant roles in tumorigenesis. Delbridge *et al.*^30^ demonstrated that BIM(*Bcl2l11*)-dependent apoptosis acts as an important tumor suppressor to prevent cancer development as a result of Rag1/2-induced DNA lesions in p53 deficient mice. Interestingly, loss of Bcl2-modifying factor did not potentiate Rag-mediated tumor development, as did loss of BIM, even though the proteins are both pro-apoptotic members of the BCL-2 family. Similarly, *Ebf1*^*+/–*^*Bcl-2*^*Tg*^ mice do not develop tumors (M. Sigvardsson, unpublished data). This observation may be due to dual inhibition of both BAX and BAK, which are direct effectors of mitochondrial apoptosis, by Bcl-x_L_, but not Bcl-2. Moreover, Bcl-x_L_ is more stable than its sister pro-survival proteins BCL-2 and Bcl2 family apoptosis regulator MCL-1.^31^ In addition, Yagi and colleagues^32^ determined that expression of dominant-negative Ikaros, found in a subset of acute myeloid leukemia patients, upregulates BCL-X_L_ but not BCL-2. Expression of BCL-2 and BCL-X_L_ is also differentially regulated during development. In mice, BCL-X_L_ expression is high in pro- and pre-B cells, as well as antigen stimulated B cells, but low in immature and resting mature B cells, while BCL-2 is highly expressed in pro-B cells and resting mature B cells.^16, 18, 32^ Mice deficient in BCL-2 exhibit normal B-cell development but lose mature cells to apoptosis.^33, 34, 35^ In contrast, BCL-X_L-_deficiency results in embryonic lethality and BCL-X_L-_deficient lymphocytes display severe defects in B-cell development, with a pronounced reduction in small (late) pre-B and immature B cells.^36^ These findings highlight the importance of apoptosis in preventing survival of neoplastic cells with DNA damage, and the lack of complete functional redundancy between anti-apoptotic factors. The collaboration of anti-apoptotic signaling pathways such as JAK/STAT or BCL-ABL activating mutations with loss of key transcription factors such as EBF1 that regulate DNA damage repair and maintain differentiation likely leads to development of aggressive, drug-resistant B-ALL in human patients.

Significantly reduced levels of *Ebf1*, *Tcf3*, *Pax5* and *Ikzf1* transcripts in unaffected EB pre-B cells allow increased transcription of targets normally repressed such as markers of other lineages (for example, NK-cell-specific genes) and early progenitors (for example, *kit*). This progenitor-like cellular milieu poises the cells for acquisition of additional defects that fully extinguish expression of lineage-determinant transcription factors and induce and/or potentiate oncogenic transformation. A similar process likely occurs in B-ALL patients, as many cancers including B-ALL express anti-apoptotic Bcl2 family members. The EB murine model of B-ALL recapitulates spontaneous generation of pre-B-cell tumors with molecular defects similar to those seen in leukemia patients with common transcription factor haploinsufficiencies, deletions or translocations, and does not depend on expression of oncogenic drivers such as c-myc and constitutively active Stat5 which are not found in most B-ALL patients. Therefore, this model may prove valuable for further study of tumorigenesis and therapy response in aggressive and/or relapsed B-ALL.

## Materials and methods

### Mice

*Ebf1*^*+/–*^mice^4^ were obtained from Y. Zhuang (Duke University, Durham, NC, USA) and previously backcrossed onto a C57BL/6J background. *Bcl-x*_*L*_^*Tg*^ mice (Tg(Emu-Bcl2L1)87Nnz)^16, 18^ were obtained from T. Behrens (University of Minnesota, Minneapolis, MN, USA) and crossed to *Ebf1*^*+/–*^mice to generate *Ebf1*^*+/–*^*Bcl-x*_*L*_^*Tg*^ mice and littermate controls. Genotyping of *Ebf1* was performed as previously described.^37^ Mice were bred and maintained in specific pathogen-free facilities at National Jewish Health and the University of Washington. All experiments were reviewed and approved by the National Jewish Health and/or University of Washington Institutional Animal Care and Use Committees.

Adoptive transfer experiments were conducted by intravenous tail vein injection of 1 × 10^5^ or 2 × 10^6^ cells isolated from spleens or lymph nodes of mice bearing primary tumors, with or without prior sub-lethal irradiation (400 rads). All tumor-bearing mice were killed upon development of clinical signs of disease. Thirty EB, 21 *Bcl–x*_*L*_^*Tg*^ and 15 *Ebf1*^*+/–*^ animals including approximately equal representation of both sexes were generated as part of this study. Animals used for analysis ranged in age from 16 to 71 weeks old, and age-matched controls were killed with each affected EB mouse. Animals were censored, according to pre-established criteria, if euthanasia was performed for humane reasons unrelated to neoplasia, such as chronic ulcerative dermatitis. No randomization was used, and no blinding was performed. Sample size estimates were based on previous experience with s.d.'s in the assays used.

### Histopathology

All histopathology was performed at The University of Washington Histology and Imaging Core. Tissues were collected, fixed in 10% neutral buffered formalin, routinely embedded and sectioned at 4 μm. Sections were stained with hematoxylin and eosin. Ki-67 (M7249, Dako, Santa Clara, CA, USA) and EBF1 (PA541632, Thermo Fisher Scientific, Waltham, MA, USA) antibodies were used for immunohistochemistry of select formalin-fixed sections from experimental mice. Antigen retrieval was performed by incubation in HIER 2 (EDTA) for 20 min at 100 °C (Ki-67) or incubation in Proteinase K for 15 min at 37 °C (EBF1), and dilutions of primary antibodies used were 1:50 (Ki-67) or 1:150 (EBF1). Three EB, three *Ebf1*^*+/–*^, and two *Bcl-x*_*L*_^*Tg*^ mice were analyzed.

### Antibodies and flow cytometry

Cell staining was performed using the following antibodies with specificities for mouse antigens: CD45R (B220) (various fluorochromes) (eBiosciences, San Diego, CA, USA; BD Pharmingen, San Jose, CA, USA; and Tonbo Biosciences, San Diego, CA, USA); IgM (various fluorochromes) (Jackson ImmunoResearch, West Grove, PA, USA); CD117 PE, CD244 PE, NK1.1 PE/APC (BD Pharmingen); CD19 eFlour450 (Tonbo Biosciences); CD335 FITC, CD24 (HSA) PE-Cy7, IgD FITC (BD Pharmingen), CD3 PcP Cy5.5, Ly-6G FITC, CD11b PE, CD25 APC, BP-1 PE, CD45.2 eFluor 450. Each individual histogram and plot shown in the figures represents data from one mouse. Overlaid histograms represent data from two mice.

### Immunoblotting

Immunoblotting was performed on whole cell extracts prepared from primary tumor cells isolated from the lymph nodes of different EB or Eμ-Myc mice, or negative selection bead-sorted B cells (mouse B cell isolation kit, 130-090-862, Miltenyi Biotec Inc., San Diego, CA, USA) from the spleen of a congenic wild-type mouse. The following antibodies were used: EBF1 (H00001879-D01P, Abnova, Taipei, Taiwan) and c-Myc (D3N8F, Cell Signaling Technology, Danvers, MA, USA). GAPDH (D16H11, Cell Signaling Technology) was used as a loading control. Each lane of the membrane shown contains tissue lysate from a different mouse.

### RNA isolation and qPCR

Bone marrow late pre-B cells (B220^+^IgM^–^BP-1^–^CD25^+^) were sorted from five wild-type and three *Bcl-x*_*L*_^*Tg*^mice using a FACSAria III (BD Biosciences, San Jose, CA, USA). Tumor cell progenitors (B220^+^CD244^+^) were isolated by FAC-sorting bone marrow of three *Rag2*^*–/–*^*Il2rγ*^*–/–*^ recipients of *Ebf1*^*+/–*^*Bcl-x*_*L*_^*Tg*^ bone marrow transfer. Tumor cells (B220^+^CD244^+^) were collected from lymph nodes of four clinically affected *Ebf1*^*+/–*^*Bcl-x*_*L*_^*Tg*^ mice. Cells were lysed in TRI Reagent (Molecular Research Center, Cincinnati, OH, USA) and RNA purified by phase separation and precipitation according to the manufacturer’s instructions. cDNA was prepared using SuperScript II reverse transcriptase (Thermo Fisher Scientific, Waltham, MA, USA) according to the manufacturer’s instructions. Quantitative PCR was performed using SYBR Green Master Mix (Thermo Fisher Scientific) and 50 nM of each primer on a Mx3005 instrument (Stratagene, Santa Clara, CA, USA). HPRT was used as a reference to calculate ΔCT for each cDNA sample in every qPCR run, and all samples were run in triplicate for each primer set. For all graphs, each point plotted represents the relative expression of the tested gene (2^^−ΔΔCT^) for a cDNA sample from one mouse. Sequences of primers used for qPCR are found in Supplementary Tables 1 and 2.

### Analysis of *Ig* rearrangements

Immunoglobulin heavy and light chain rearrangements were performed as previously described.^37^ Briefly, DNA was purified from lymph nodes or spleens, PCR was performed, and amplification products were resolved by agarose gel electrophoresis for detection of heavy chain rearrangements. Ig lambda rearrangements were detected using PCR with AmpliTaq DNA polymerase (Thermo Fisher Scientific) and a cocktail of primers specific for Vλ1/Vλ2 (5′-AATCTGCACTCACCACATCACCTGGTG-3′) and antisense primers corresponding to introns of J-Cλ1 (5′-ATCTCTTTTAGCCCCCTGTG-3′), J-Cλ2 (5′-CAGAGAGAAAAAAGACCCTTGC-3′) and J-Cλ3 (5′-CAGAGAGATAAATGACCCTTGC-3′). Amplification was followed by phenol:chloroform extraction of PCR products, desalting on G50 spin columns, and sequential restriction enzyme digestion with *Bsr*D1 and *Hin*dIII (New England Biolabs, Ipswich, MA, USA). Each lane of the gels shown contains DNA from a different mouse.

### Sequencing of *Ebf1* genes

DNA was purified by proteinase K digestion, phenol/chloroform extraction and ethanol precipitation of paired tumor (lymph node or spleen) and normal tissue (tail or ear clip) samples from seven clinically affected *Ebf1*^*+/–*^*Bcl-x*_*L*_^*Tg*^ mice, one unaffected *Ebf1*^*+/–*^*Bcl-x*_*L*_^*Tg*^ mouse, and B220^+^CD244^+^ FACS-sorted bone marrow from three healthy and one affected *Ebf1*^*+/–*^*Bcl-x*_*L*_^*Tg*^ bone marrow transfer recipient mice. A custom SureSelect-XT DNA kit (Agilent, Santa Clara, CA, USA) was used to sequence *Ebf1* genes using a MiSeq instrument (Illumina, San Diego, CA, USA). UCSC mm9, NCBI Build 37 was used as the basis for probe design in the kit and was also used as the alignment reference. The control-FREEC application was used to estimate copy number gain and loss across the *Ebf1* gene in tumor vs normal samples, and the Integrative Genomics Viewer (Broad Institute, Boston, MA, USA) was used to visualize sequence coverage across the gene.

### Statistical analyses

All statistical analyses were performed using GraphPad Prism version 6.01 for Windows, GraphPad Software, La Jolla CA, USA. A log-rank (Mantel–Cox) test was performed to compare tumor-free survival curves in Figure 1a. For all qPCR results (Figure 4b, Supplementary Figures S4–S6), Student’s unpaired two-tailed *t*-tests with equal variance were performed. Center values shown on graphs represent means and error bars represent s.d.'s, and individual values are plotted for all groups with *N* (biological replicates) less than or equal to five. An F-test to compare variances was performed on each data set. For some transcripts examined (for example, *CD244* and *kit*) EB progenitor and/or tumor cells exhibited higher variance between samples than controls. Correlations between gene expression microarray data (Figure 5d) were performed using Pearson *r* with two-tailed *p*-values.

## Figures and Tables

**Table 1:** EB leukemia cells display a pre-B-cell surface phenotype

*Mouse #*	*B220*	*CD19*	*CD117*	*BP-1*	*CD25*	*lgM*	*CD3*	*Ly-6G*	*CD11b*	*CD335*	*NK1.1*	*CD244*
EB 15	+	+	+	+	ND	−	−	ND	ND	ND	+	+
EB 39	+	+	−	+	ND	−	−	ND	ND	ND	−	+
EB 54	+	+	lo	+	ND	−	−	ND	ND	ND	+	+
EB 87	+	+	+	+	ND	−	−	ND	ND	ND	+	+
4606	+	−	+	−	+	−	−	−	−	−	lo	+
5132	+	+	+	−	+	−	−	−	−	−	lo	+
K832	+	+	ND	−	+	−	−	−	−	−	lo	+

lo, low surface expression, but higher than negative controls; ND, not determined. The table lists cell surface markers present or absent on six different primary tumor isolates from *Ebf1*^*+/–*^*Bcl-x*_*L*_^*Tg*^mice and one (K832) which spontaneously developed in an *Ebf1*^*+/–*^*Bcl-x*_*L*_^*Tg*^bone marrow transfer, as determined by flow cytometric analysis.

**Table 2:** EB leukemia cells transfer disease through multiple hosts

*Transfer experiments*	*1st*	*2nd*	*3rd*	*4th*	*5th*	*6th*	*7th*	*8th*	*9th*
EB 76	8/8	6/6	4/4	4/4	4/4	3/3	3/3	3/3	
EB 54	5/5	4/4	4/4	4/4	4/4	6/6	3/3	2/2	
EB 39	5/5	4/4	4/4	4/4	4/4	4/4	4/4	4/4	2/2
EB 15	4/4	4/4	4/4	4/4	3/3	3/3	2/2		
EB 19	4/4	4/4	2/2	2/2					
EB 87	4/4	3/3	3/3	3/3					

Six different *Ebf1*^*+/–*^*Bcl-x*_*L*_^*Tg*^primary tumor cell isolates were intravenously injected into sublethally irradiated *Rag1-*deficient recipient mice. Tumors were serially transferred through 4–9 hosts depending on the isolate. Numbers represent animals killed due to disease development with respect to the total number of recipients in the cohort (for example, 4/4=4 mice developed clinical disease out of 4 mice injected).
