# Supplementary material for: Spontaneous loss of B lineage transcription factors leads to pre-B leukemia in Ebf1+/–Bcl-xLTg mice
Source: Oncogenesis. 2017 Jul 10;6(7):e355–. doi: 10.1038/oncsis.2017.55 (PMC5541707; doi:10.1038/oncsis.2017.55)
Supplement: Supplementary Information [file oncsis201755x1.docx]

**Supplementary Information**


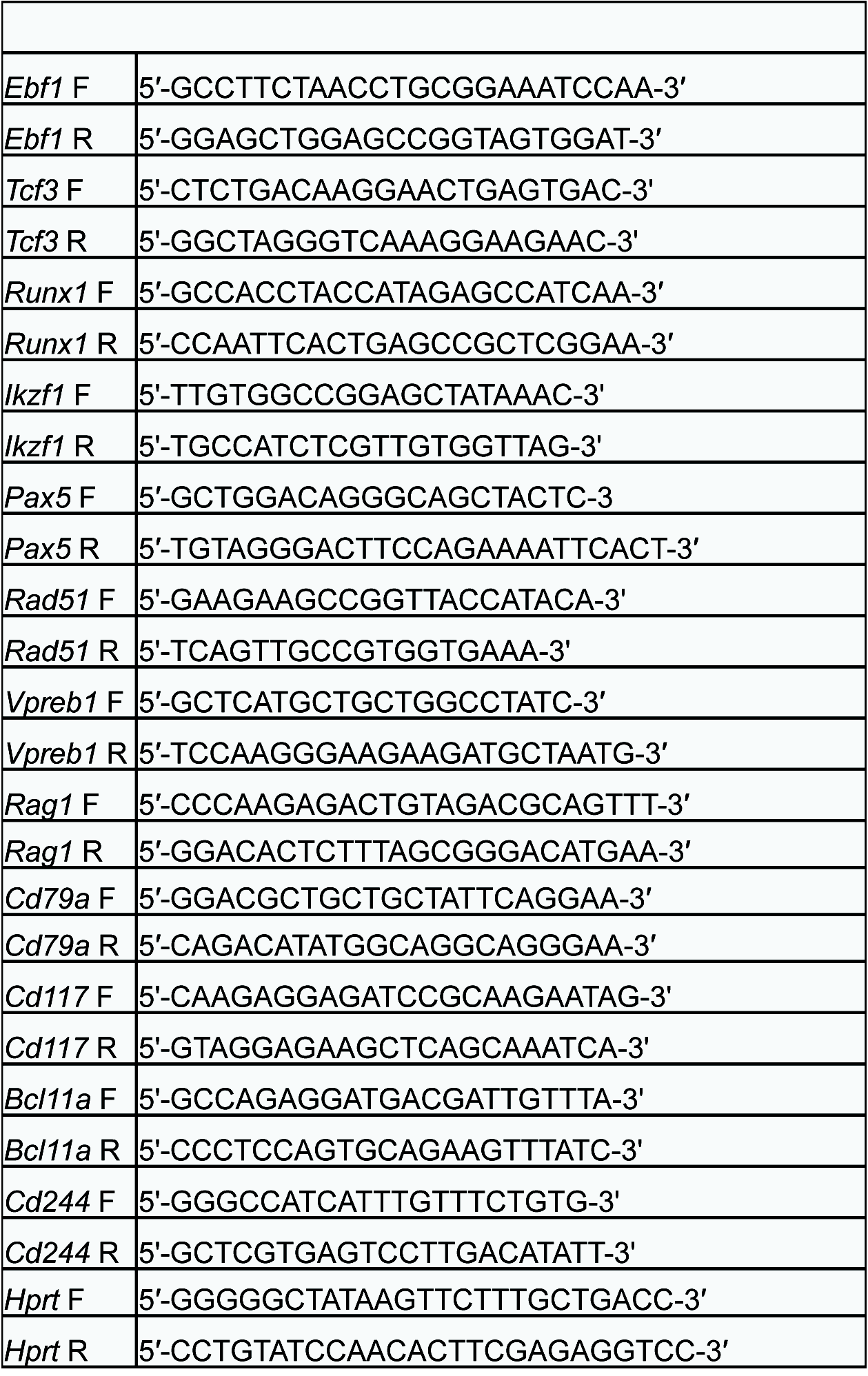


**Table S1. qRT-PCR primer sequences.** Sequences of primers used in qRT-PCR in

Figures 4 and S4.


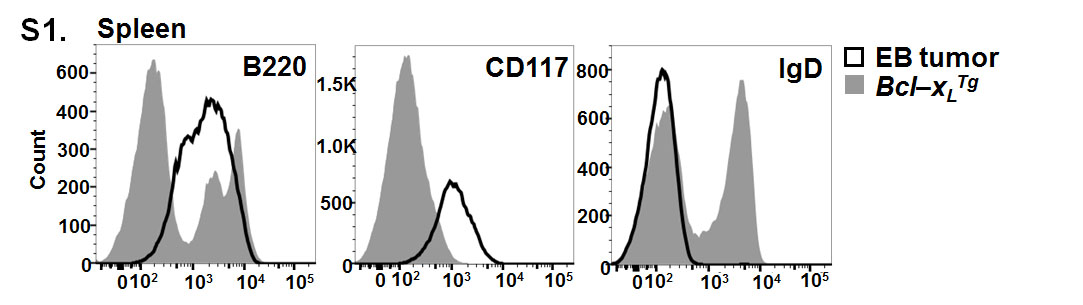


**Figure S1.** **EB tumor cells display a precursor B phenotype not present in the periphery of aged *Bcl-x_L_^Tg^* control mice.** Histograms compare cell surface expression of selected markers from EB vs. aged *Bcl-x_L_^Tg^* littermate control splenocytes. Histograms are representative of 4 EB and 3 *Bcl-x_L_^Tg^* control mice analyzed.

**
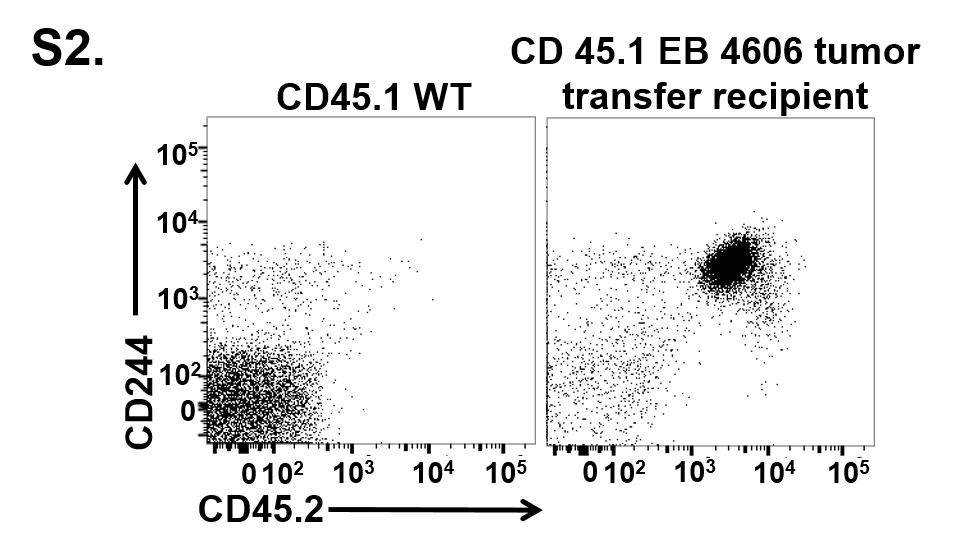
**

**Figure S2. EB tumor cells are highly malignant.** Primary tumor cells from *Ebf1^+/–^Bcl-x_L_^Tg^* mice (CD45.2+) were transferred to non-irradiated, immunocompetent CD45.1 recipient mice by intravenous injection of 1 x 10^5^ cells. This mouse was representative of four recipient mice of two different primary tumors. The right dot plot shows accumulation of CD45.2+/CD244+ cells in the spleen of a CD45.1 recipient. All tumor cell recipient mice were euthanized due to clinical disease development.


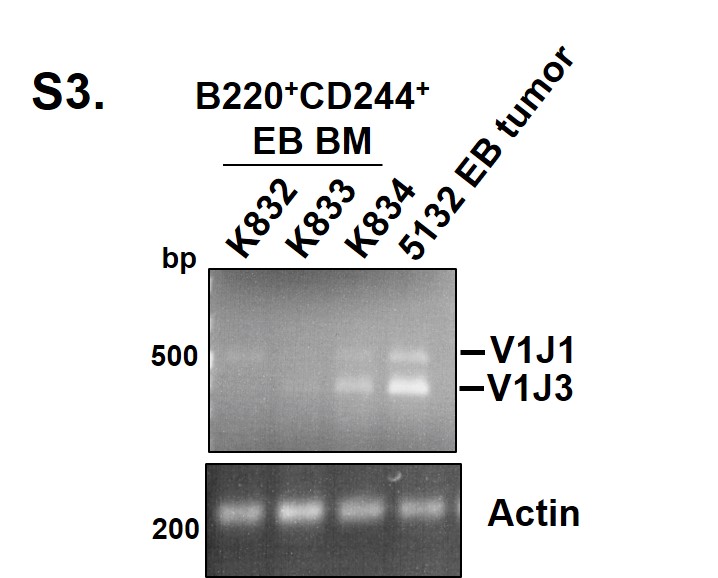


**Figure S3. The B220^+^CD244^+^ population in EB bone marrow contains immunoglobulin lambda light chain rearrangements.** DNA was purified from sorted B220^+^CD244^+^ bone marrow from three *Ebf1^+/–^Bcl-x_L_^Tg^* bone marrow transfer recipients and one EB tumor, and PCR was used to detect immunoglobulin lambda rearrangements.

**S4.**

**
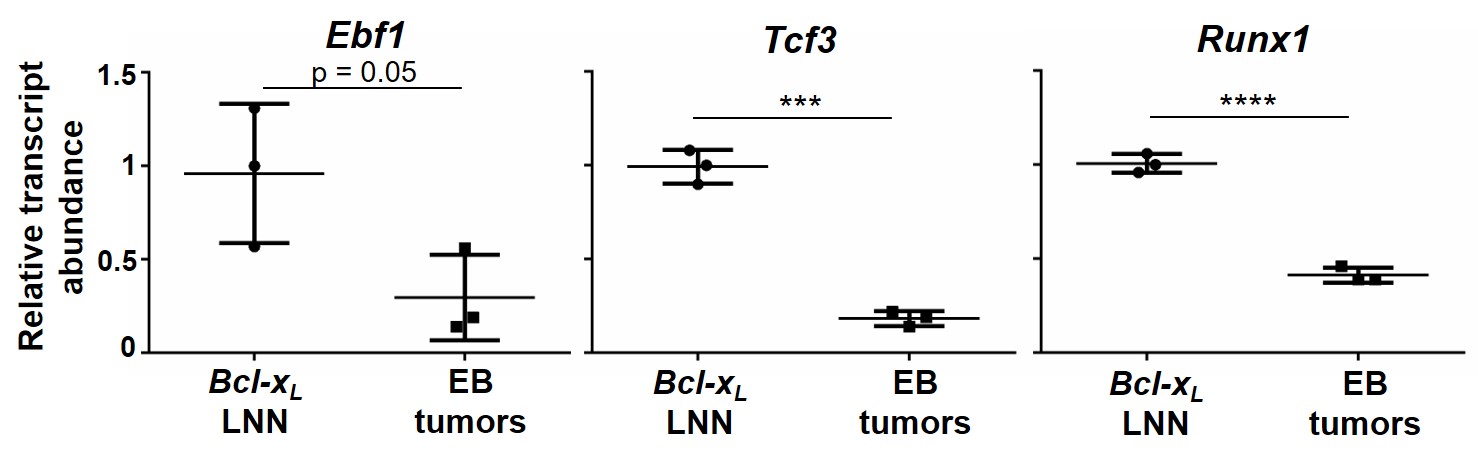
**

**Figure S4. Expression of transcription factors is reduced in EB tumors compared to *Bcl-x_L_^Tg^* control lymph nodes.** RT-qPCR analysis of relative transcript levels from selected genes in whole lymph nodes from *Bcl-x_L_^Tg^* control mice (n=3) and leukemic cells from EB mice (n=3). p <0.05 = *, p <0.01 = **, p<0.001 = ***, p<0.0001 = ****, ns = not significant (p>0.05). Primers used for qRT-PCR are in Table S1.

**
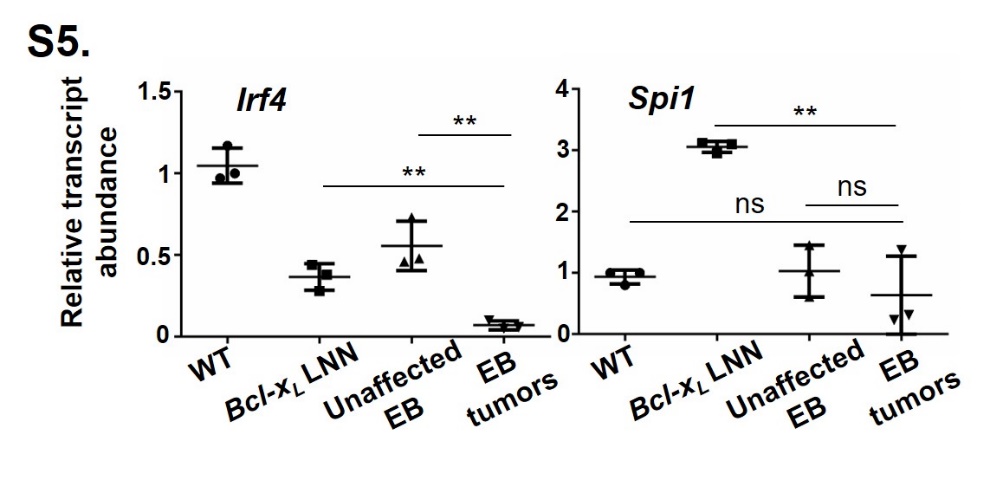
**

**Figure S5. Expression of *Irf4*, but not *Spi1*, is reduced in EB tumors.** RT-qPCR analysis of relative transcript levels from selected genes in sorted late pre-B cells (B220^+^IgM^–^BP-1^–^CD25^+^) from the bone marrow of congenic wild-type mice (n=3), lymph nodes from *Bcl-x_L_^Tg^* control mice (N=3), B220^lo^CD244^+^ (presumed tumor progenitor) cells from the bone marrow of healthy EB bone marrow transfer recipients (Unaffected EB, n=3), and leukemic cells from EB mice (EB tumors, n=3). p <0.05 = *, p <0.01 = **, p<0.001 = ***, p<0.0001 = ****, ns = not significant (p>0.05). Primers used are in Table S2.

**
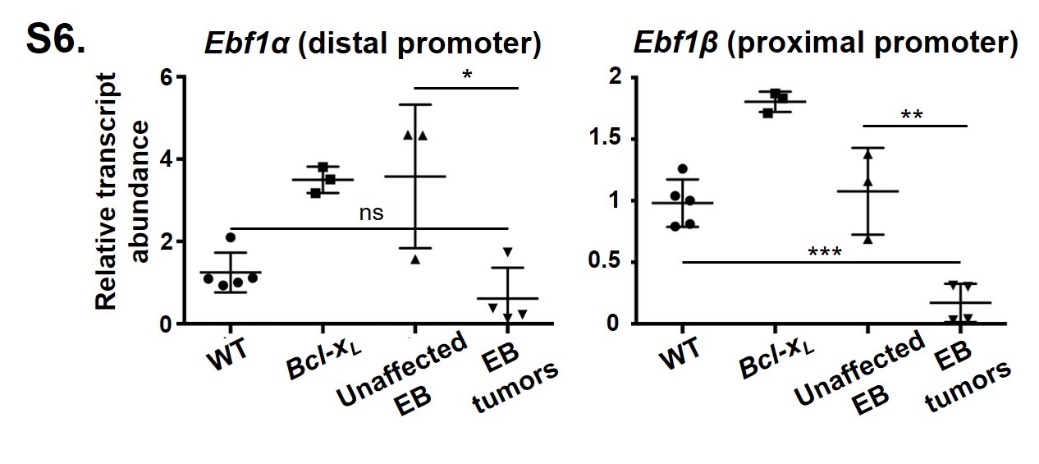
**

**Figure S6. Expression of the proximal *Ebf1β* promoter is reduced in EB tumors.**

RT-qPCR analysis of relative transcript levels from two *Ebf1* promoters in sorted late pre-B cells (B220^+^IgM^–^BP-1^–^CD25^+^) from the bone marrow of congenic wild-type mice (N=5) or *Bcl-x_L_^Tg^* control mice (N=3), B220^lo^CD244^+^ (presumed tumor progenitor) cells from the bone marrow of healthy EB bone marrow transfer recipients (Unaffected EB, N=3), and leukemic cells from EB mice (EB tumors, N=3). p <0.05 = *, p <0.01 = **, p<0.001 = ***, p<0.0001 = ****, ns = not significant (p>0.05). Primers used are in Table S2.

|  |  |
| --- | --- |
| *Irf4* F | 5'-GGCAAGCAGGACTACAATCGTG-3' |
| *Irf4* R | 5'-TTGGCTCCCTCTGGAACAATCC-3' |
| *Spi1* F | 5'-CGGATGTGCTTCCCTTATCAAAC-3' |
| *Spi1* R | 5'-TGACTTTCTTCACCTCGCCTGTC-3' |
| *Ebf1α* F | 5'-CAGAGGGCCTTTGAGCTTAGG-3' |
| *Ebf1α* R | 5'-CTTTCTTGCCAGAGGTAGCTGC-3' |
| *Ebf1β* F | 5'-CCCTCCTTGTTATCGCTCAGTT-3' |
| *Ebf1β* R | 5'-TTGAGTCGATGAGACTCGCG-3' |

**Table S2.** Sequences of primers used for qRT-PCR in Supplementary Figures 5 and 6.
